# Perforated Typhoid Ulcer
1Read before the Bristol Medico-Chirurgical Society, January 14th, 1903.


**Published:** 1903-03

**Authors:** H. G. Kyle

**Affiliations:** Assistant-Surgeon to the Bristol General Hospital.


### PERFORATED TYPHOID ULCER.[1]

BY

H. G. KYLE, M.B., B.Ch. Oxon.,

*Assistant-Surgeon to the Bristol General Hospital.*

THE subject of perforation in typhoid fever is one of perennial interest both to the physician and the surgeon. Upon the early recognition of its occurrence by the physician depends the success of interference by the surgeon.

It is a field in which the surgeon has been more often baffled than victorious, and one in which he has but a limited opportunity of enlarging, and profiting by, his own personal experience; for perforation is not a condition very frequently met with, as it occurs in only 2 to 5 per cent. of all cases. Probably about fifty cases pass through each of our Bristol hospitals in the year: this means an expectation of from one to three perforations; and these, divided between four or five surgeons, render the chances considerably against any individual surgeon having to deal with a single case in the course of the year.

Such being the case, we are principally dependent on the study of the cases of others for the advancement of our

[1] Read before the Bristol Medico-Chirurgical Society, January 14th, 1903.

knowledge in the methods of diagnosing and dealing with this condition. And for these reasons I think the details of a case I had recently under my care will be of some interest, and perhaps profit, to record:

The patient, a strong, healthy man, æt. 27, was admitted into the Bristol General Hospital, under the care of Dr. Odery Symes, on August 29th. His temperature was then 104°; he had been ill for a week, but had not stopped work. On September 3rd he was seized with severe abdominal pain at six o'clock in the evening, and had a rigor; the pain got better later on, but returned at intervals until midnight; it was not so severe again as at first, and he had intervals of sleep between the attacks of pain.

I saw him, with Dr. Symes, at 2 a.m. on September 4th. He was not then complaining of much pain; he was lying on his right side, and did not appear to be in any pain or distress. His colour and general appearance were good; pulse, 100, good quality; temperature, 101°; the abdomen was distended, but tympanites had been marked earlier in the case; the liver dulness was absent; the abdomen moved universally with respiration, but the movements were somewhat limited, and more markedly so in the right lower part; there was some fixed tenderness about McBurney's point, not very acute; also a little general tenderness; he was sweating freely.

In view of the sudden onset of intense pain, together with the persisting though slight tenderness, and absence of liver dulness, I advised an exploratory operation, although there was no collapse nor increase in pulse frequency.

I opened the abdomen by an oblique incision over the right iliac fossa. On incising the peritoneum, about eight ounces of turbid yellow fluid escaped under some pressure; this collection appeared to be almost shut in by the neighbouring coils of intestine, between which, however, there were no adhesions. The coils of gut were injected; but there was no deposit of lymph on them, except in the immediate neighbourhood of the perforation, which was easily found at the lower end of the ileum. The hole was about the size of a goose-quill, and situated in the centre of a much-thickened patch; it was partially covered by a process of adherent omentum, from beneath which a small amount of intestinal contents was escaping. I cleaned the intestine in the neighbourhood with sponges; also the pelvis, in which there was some turbid fluid. I did not find any neighbouring patches which showed signs of perforating.

On attempting to close the perforation by folding in the whole ulcer, I found it impossible to do so, as the induration involved so much of the circumference of the bowel; sutures passed through the indurated part cut out immediately when tied. It was found necessary, therefore, to close the hole by lightly-tied sutures, and leave the sutured part under the wound with a gauze drain. A tube was passed into the pelvis.

The patient was no worse for the operation; his temperature, taken a few hours later, had dropped to 97.4°, and about twelve hours afterwards began to ascend again to its former level. No symptoms of peritonitis developed. There was free discharge from the wound for ten days, when he died from the continuance and severity of the typhoid fever.

*Post-mortem* examination showed that no peritonitis had developed, nor had any second perforation either threatened or occurred. The diseased portion of gut was adherent to the abdominal wound around the base of the ulcer, which was sloughy; and a fæcal fistula was present, although no actual fæcal matter had so far been observed in the discharges.

The result of operation in this case was successful, in so far that the patient was saved from peritonitis and death from the effects of perforation. Had he not died eventually from the progress of his disease, he would presumably have recovered with a fæcal fistula, which would have closed, or could have been dealt with later on.

Some recent statistics emphasise very forcibly the importance of early interference. The recoveries after operation undertaken during the first twelve hours were 40 per cent.; during the second twelve hours, 10 per cent.; and after twenty-four hours no cases recovered.

To lower the hitherto great mortality, we must therefore operate within the first few hours; and the question here presents itself, What are the indications for operation in these first few hours? That this is not an easy one to answer is shown by the number of cases that have been left until too late, by our own experience when the question has had to be answered by the bedside, and by the way it is treated of in the leading works on medicine. On consulting these, we learn practically nothing about the early indications. We are told of sudden acute pain and tenderness, collapse, sudden fall of temperature and the symptoms of general peritonitis are given in detail; but these are of no use for the purposes of the surgeon. Judging from the symptoms described in the recorded successful cases, I think we may say that the only constant early symptom is the sudden onset of pain, with localised and persisting tenderness; but unfortunately pain and tenderness occur apart from perforation, and it is often far from easy to differentiate between the pain of colic and that of perforation.

The surgeon is here much handicapped, in that, since he does not have the opportunity of observing and becoming familiar with the ordinary course of the disease, he is not so well fitted to make this early differentiation. It may be said, as a rule, that the pain of colic tends more to move about, to be less persistent, and is less likely to be associated with tenderness. In my case the pain was very acute at first, recurred at intervals, and was accompanied with tenderness, which, though slight, was persistent and localised.

Another symptom which is generally present early is some fixity of the abdomen: this was present in a slight degree in my case. The combination of pain, tenderness, and some localised fixity will, I believe, be found to be a constant indication for exploration.

The next consideration is the pulse: there is generally a rise in the frequency, which continues to increase. This is a valuable indication, but was absent in my case.

Absence of liver dulness is of undoubted value in cases where it is first noted at the onset of other symptoms; but to make this symptom of value, it should be a routine practice to estimate the line of liver dulness daily, especially in cases associated with tympanites.

Collapse, one of the classical symptoms of perforation, if absent, must not be waited for: occurring early, it generally indicates a large perforation, with extensive extravasation into the abdomen. In my case the sudden drop in temperature, which may have represented collapse, did not occur until several hours after the operation. When the perforation is small, there is not much extravasation at first, and what does escape probably remains localised for a few hours until the collection has so increased in quantity that it suddenly displaces the coils of intestine that held it confined, and is rapidly diffused through the peritoneal cavity. This would, I believe, have been the sequence of events in my case, had it been left. On opening the abdomen, there was a collection of about eight ounces of fluid, apparently localised, and contained between coils of intestine without any adhesions: had the pressure become much greater, this collection would doubtless have pushed aside the protecting coils of gut and become diffused through the belly cavity. This occurrence would in all probability have been attended by signs of collapse; but we must endeavour to anticipate such an event.

Sweating—not the cold, clammy sweat of collapse, but a profuse perspiration—occurred in my case, and has been noted in others. I cannot say how often it occurs, but some observers lay stress on this as an early symptom. Sweats undoubtedly occur at times in some cases of typhoid, but they are the exception: it is therefore a symptom to be observed.

A rigor occurred at the commencement of the symptoms in my patient, and has been noted in other cases; but rigors from time to time are a feature of some attacks of typhoid: perhaps an isolated rigor, in a case where rigors have not occurred before, may be of some significance.

Vomiting has been noted as a symptom; but is, I believe, rare apart from peritonitis.

From the foregoing considerations, I think we may say that if we are to anticipate the graver complications of perforation by early surgical interference, we may take, in the present state of our knowledge, the following indications as demanding operation: Pain, localised tenderness, and fixity of part of the abdomen. The severity of the pain and tenderness will vary with the condition of the patient: the worse his condition, and the more apathetic he be, the less marked will be his symptoms. We shall probably be able to elicit some one or more of the other symptoms enumerated above, and, taken in conjunction with these three principal indications, they will afford us valuable evidence.

I call these, not symptoms of perforation, but indications for operation; for, as a rule, we cannot positively diagnose a small perforation so early after its occurrence. All that we can say in many cases is, that the symptoms are sufficiently suspicious to justify an exploratory incision. If we act on these principles, we shall doubtless on some occasion open an abdomen needlessly; but if we do, the risk to the patient is small, and not to be compared with the risks of an undetected perforation. It is, moreover, a procedure by which we shall not lose any lives, but probably be enabled to save many. Apart from perforation, I believe that pain and tenderness often indicate a local peritonitis over the base of an ulcer, which in the end, however, may escape perforation; but the risk of perforation in such a case must be very great, and by an early exploration we shall be able to anticipate and prevent the occurrence of perforation.

The anæsthetic has been urged as a serious objection, especially when pulmonary complications exist; but an anæsthetic, though desirable, is not necessary. A considerable number of exploratory incisions have been made, especially by American surgeons, under local anæsthesia, and ulcers have been stitched up, with very little discomfort to the patient, with aid of eucaine for the skin incision.

We can considerably lower the present mortality of these cases; but we shall never obtain a very high percentage of recoveries, because of the very nature of the cases with which we are dealing. To begin with, typhoid itself has a mortality of 10 per cent. or over; then perforations in patients already suffering from profound toxæmia pass unobserved—and even were they detected, the patients are beyond the aid of surgery. Large perforations and extensive fæcal extravasation must nearly always be fatal.

Multiple and recurrent perforations add considerably to the difficulties; but our methods of dealing with these are probably not yet perfect. The practice at present, in cases where the ulcer cannot be effectually closed by suture, is to be satisfied with the chances of a fæcal fistula or to establish an artificial anus. The question of resection must always present itself in these cases, especially in cases where there is a considerable length of bowel much infiltrated, more than one perforation, and perhaps several areas where perforation seems imminent. Packing and isolation of so large an area seems almost hopeless, though such a case [1] has been recorded where the cæcum and large part of the ileum were badly affected. After packing off

[1] *Ann. Surg.*, 1901, xxxiii. 645.

all the diseased bowel from the rest of the peritoneum, the patient recovered with a fæcal fistula.

Resection means generally a more or less prolonged operation, and addition to the shock, and rapidity of operating is of prime importance in these cases. But I believe that, by our improved technique and more rapid methods of resection, we could, in many cases perhaps, more rapidly resect a portion of bowel than stitch up several ulcers and deal with one or more areas of impending perforation,—a procedure perhaps, after all, to be of no avail if more diseased areas give way later on.

Perhaps the greatest argument against resection is, that in many cases it must involve a considerable portion of the ileum and the cæcum, because one would have to get beyond the area of ulcerations. No successful cases of resection have been recorded so far; but when we have so improved our powers of diagnosis that we can get the cases early, we shall be in a better position to improve our methods of dealing with the lesions found.

Our present position as regards the question of resection is probably this: Most cases can be satisfactorily dealt with by suture, many cases are too bad to attempt it, but there is probably a certain percentage of cases in which it offers the best chance. But we can say nothing from experience on this point, as the method has not had a trial; but I believe that, by getting suitable cases sufficiently early, we shall feel ourselves justified in giving it a trial.

The discussion on this paper is reported on page 88.

